# Same‐Day Discharge After Transcatheter Aortic Valve Implantation: Who, When, and How?

**DOI:** 10.1002/ccd.70715

**Published:** 2026-07-01

**Authors:** Hilal Khan, Debbie Stewart, Sarah Lamb, Rajiv Das, Richard Edwards, Timothy Cartlidge, Mohammad Alkhalil, Azfar Zaman, Mohamed Farag

**Affiliations:** ^1^ Cardiothoracic Department Freeman Hospital Newcastle Upon Tyne UK; ^2^ School of Health, Medicine and Life Sciences University of Hertfordshire Hertfordshire UK

**Keywords:** aortic stenosis, day‐case TAVI, same‐day discharge, transcatheter aortic valve implantation

## Abstract

The burden of severe aortic stenosis requiring treatment is growing due to ageing populations. This places increased demands on health resources due to the growing number of transcatheter aortic valve implantation (TAVI) procedures and hospital bed occupancy. There are several studies of same‐day discharge (SDD) after coronary angioplasty demonstrating its safety, particularly in transfemoral cases. There has been significant evolution in techniques and technologies underpinning TAVI, which have reduced its risk profile over time. As a result, several observational studies have demonstrated that SDD after TAVI is safe and effective. Careful selection of low‐risk patients and a minimalist TAVI approach, with appropriate post‐procedure review and follow‐up, are crucial for successful SDD after TAVI. However, there is a need for randomised trials to validate this practice. This article provides a state‐of‐the‐art overview and guidance to safely and effectively implement a SDD‐TAVI service to improve the healthcare system efficiency while enhancing patient satisfaction and recovery.

## 1 Introduction

Severe aortic stenosis represents a growing health burden due to the phenomenon of progressively ageing populations observed globally, especially in Western nations [1]. Surgical aortic valve replacement remains the mainstay of treatment for younger patients under 70 years of age due to uncertainty about the durability of transcatheter aortic valves [2]. However, the majority of patients requiring intervention are over 70 years of age, and in this cohort, transcatheter aortic valve implantation (TAVI) is the recommended treatment modality [2, 3].

The growth in TAVI follows sequential trials in high‐, intermediate‐, and low‐risk patients, supporting its utility across these groups, with the exception of the young [4, 5, 6]. When outcomes are deemed non‐inferior to those of surgical valve replacement, TAVI is often preferred for its minimal invasiveness and shorter recovery time. Consequently, there has been a significant increase in TAVI procedures with an anticipated annual growth rate of 4%−10% [7]. This has already placed considerable pressure on waiting times, with many patients failing to meet the 18‐week target recommended in the United Kingdom [8]. The “Valve for Life” initiative has proposed an even shorter, cancer‐like fast‐track pathway with a target of 8 weeks from referral to treatment to address the high mortality associated with delays [9]. Whilst these targets are not yet consistently achieved, centres are implementing algorithms, such as the SWIFT TAVI algorithm, to identify and prioritise high‐risk patients at the expense of longer waiting times for lower‐risk patients [10]. The need for more efficient use of resources has led to recommendations for expedited discharge and the minimisation of hospital bed utilisation [8]. The next pivotal moment in the evolution and delivery of TAVI services is a shift in the standard of care toward safe, same‐day discharge (SDD).

Guidance for the transition to SDD can be drawn from percutaneous coronary intervention (PCI), which underwent a similar evolution in the early 2000s. Several studies comparing SDD with overnight stay in patients with both acute and chronic coronary syndromes have been performed [11, 12, 13, 14, 15, 16, 17, 18, 19, 20, 21, 22]. These studies, including those carried out via the femoral artery using 8 French sheaths, demonstrated the safety of vascular closure devices, early mobilisation, and SDD, with no increase in bleeding/vascular complications or in major adverse cardiovascular events (Table 1) [23]. Furthermore, most complications related to the procedure occur either within 6 h or after 24 h, and those occurring within 6 h are detected before hospital discharge. In contrast, those occurring after 24 h are not detected by routine next‐day discharge [22, 23].

**Table 1:** Randomised controlled trials of same‐day discharge after percutaneous coronary intervention.

Study/year	Number of centres	Sample size	Access	Sheath size	Bleeding/vascular complications (No)	Vascular closure device (%)	Post‐PCI monitoring period (hours)	MACE (No)
Knopf et al. [11]	1	SDD 43 ONS 47	Transfemoral	8 French	0 1	0 0	8–10 h	0 1
Carere et al. [12]	1	SDD 50 ONS 50	Transfemoral	8 French	2 0	100 0	8 h	0 0
Bertrand et al. [13]	1	SDD 504 ONS 501	Transradial	5/6 French	28 22	0 0	4–6 h	7 9
Heyde et al. [14]	1	SDD 403 ONS 397	Transfemoral	5/6 French	43 41	0 0	4 h	11 17
Glaser et al. [15]	1	SDD 19 ONS 20	Transfemoral	NR	0 0	100 100	2 h	0 0
Falcone et al. [16]	1	SDD 23 ONS 21	Transfemoral	NR	0 1	100 100	3 h	0 0
Kim et al. [17]	2	SDD 150 ONS 148	Transfemoral	NR	2 3	100 100	6 h	0 1
Dagdelen et al. [18]	1	SDD 65 ONS 65	Transradial	NR	1 0	0 0	6 h	0 0
Dhakam et al. [19]	1	SDD 110 ONS 110	NR	5/6 French	0 0	NR	NR	1 0
Clavijo et al. [20]	2	SDD 50 ONS 50	Transfemoral	5−8 French	1 0	100 100	6 h	2 1
Malik et al. [21]	1	SDD 50 ONS 50	Transradial	NR	3 4	0 0	6 h	0 0
Bogale et al. [22]	1	SDD 38 ONS 44	Transradial	NR	0 0	0 0	6 h	0 0

Abbreviations: MACE, major adverse cardiovascular events; NR, not reported; ONS, overnight stay; PCI, percutaneous coronary intervention; SDD, same‐day discharge.

The experience in coronary intervention provides a useful framework for TAVI. Although early studies in PCI demonstrated the safety of early mobilisation after vascular closure devices [23], this is only one of several considerations in the management of patients after TAVI. Two additional factors may influence the safety of discharge after TAVI: conduction system disturbances and the less frequent valve‐related problems, such as late valve migration, significant paravalvular leak, or delayed coronary obstruction. Studies on conduction disturbances note a similar trend within 6 h or after 24 h of the procedure [24]. Similarly, valve‐related complications either occur peri‐procedurally or many days to months after the index procedure [25]. Thus, this sets the scene for exploring the option of SDD in the TAVI setting.

### 1.1 The Evolution of the TAVI Procedure

The initial TAVI procedures in the early 2000s were performed via the transapical approach and came with a significant in‐hospital mortality of some 10% [26]. The first transfemoral TAVI was successfully carried out in a man in 2005 [27]. Studies have shown a substantial reduction in both in‐hospital and 1‐year mortality among patients treated with the transfemoral approach [28]. The initial PARTNER trial reported the use of a 22‐ or 24‐French sheath to deliver the valve [4]. Improvements in technologies have enabled the development of smaller‐profile valves, allowing delivery via a 14‐16 French sheaths in the more recent PARTNER 3 trial [6]. This incredible progress in safer access and the miniaturisation of sheath size has contributed significantly to reductions in procedural mortality and vascular complications [29].

Furthermore, the initial TAVI procedures were performed under general anaesthesia and required trans‐oesophageal echocardiography to guide valve sizing and deployment. The development of standardised computed tomography (CT) analysis protocols for planning TAVI procedures revolutionised the selection of valve size, type, and vascular access. It also improved the prediction of potential complications, such as coronary obstruction and aortic annular rupture [30, 31]. The shift toward conscious sedation has further enhanced patient outcomes and reduced procedural complications [32]. In addition, it has enabled earlier mobilisation and discharge compared with patients undergoing general anaesthesia [33].

Extensive registry data have showcased the evolution of the TAVI procedure, with improvements in operator experience, procedural techniques, and equipment design that have translated to a significant reduction in post‐procedural complications [34]. One study demonstrated a significant decrease in in‐hospital death from 4.42% in 2012 to 0.84% in 2019 [35]. This was paired with substantial reductions in the need for pacemaker implantation and vascular complications [34, 35]. Ongoing improvements in prosthesis design will further refine valve performance, reducing the risk of vascular complications by further decreasing the valve profile and sheath size. Adjustment of the prosthesis sealing skirt may further reduce the rate of paravalvular leak and the risk of pacemaker implantation [36]. The evolution of operator experience and equipment has also increased confidence in using single‐access and/or zero‐ or ultra‐low‐contrast angiography in suitable patients, further reducing the risk of procedural complications, including contrast‐induced nephropathy, and enhancing the minimalist TAVI approach [37, 38, 39].

**Figure 1:**
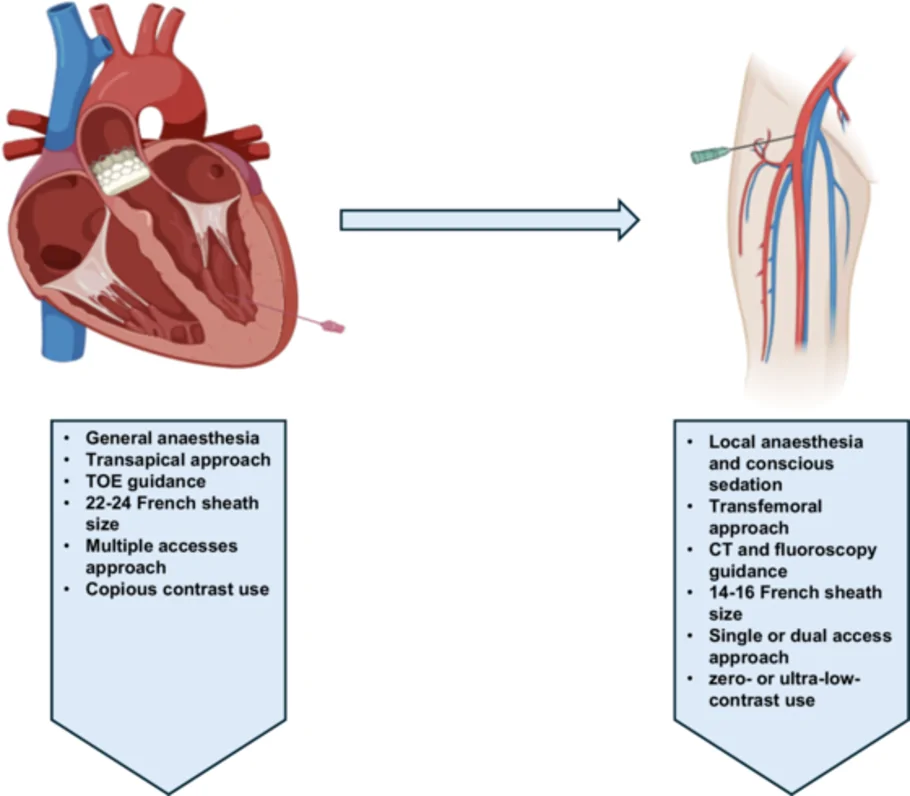
Evolution of the TAVI procedure. [Color figure can be viewed at wileyonlinelibrary.com]

The contemporary TAVI procedure, after numerous iterations, is considerably lower risk than it was at its inception over two decades ago (Figure 1). Recognition of this landmark moment in TAVI is necessary to bring about a change in practice from managing TAVI as a high‐risk procedure to a routine day‐case procedure with the adoption of SDD.

### 1.2 Current Evidence for SDD After TAVI

The first reported case of TAVI SDD was performed a decade ago in a low‐risk patient after a transfemoral approach via a 20 French sheath. The patient was mobilised after 6 h and monitored for a total of 10 h before hospital discharge, with no complications at 30‐day follow‐up [40]. Further interest in this has been fuelled during the COVID pandemic. Thereafter, a few small observational studies have demonstrated the safety and feasibility of SDD following transfemoral TAVI (Table 2) [49]. Four studies reported the use of two suture‐mediated ProGlide systems for percutaneous closure [41, 44, 46]. Four studies reported routine use of protamine sulfate to reverse unfractionated heparin, aiming to improve haemostasis, shorten procedure times, and reduce hospital stays [40, 41, 43, 45, 46, 50]. The benefits of protamine in establishing safer early mobilisation post‐femoral venous access have been well established in the context of radiofrequency ablation procedures [51]. Most studies advocated early mobilisation within 4−6 h of femoral vascular closure, and none of the patients exceeded an 8‐h monitoring period (Table 2). Early mobilisation after vascular closure devices in transfemoral PCI cases has been well validated for safety [12]. Concerns regarding the safety of early mobilisation with large‐bore vascular access after TAVI have primarily been addressed by several observational studies [25, 41, 42, 43, 44, 45, 46, 47, 48].

**Table 2:** Studies of same‐day discharge after transcatheter aortic valve implantation.

Study/year	Sample size	Access	Permanent pacemaker in‐situ (%)	Valve type	Bleeding/vascular complications (No)	Vascular closure device (%)	Protamine use (%)	Post‐TAVI monitoring period (hours)	All‐cause mortality (No)
Williams et al. [41]	SDD 13	TF	100%	46% BEV	0	100% ProGlide	100%	7 h	0
Okoh et al. [42]	SDD 13	TF	0%	100% BEV	0	NR	NR	6 h	0
Perdoncin et al. [43]	SDD 29 NDD 128	TF	10% 9%	83% BEV 93% BEV	0 1	NR	96.6% 78.1%	4 h	0 0
Pop et al. [44]	SDD 29 NDD 84	TF	38% 7%	97% BEV 98% BEV	0 3	100% ProGlide	NR	8 h	0 1
Krishnaswamy et al. [45]	SDD 114 NDD 329	TF	13% 13%	91% BEV 85% BEV	7 36	NR	100% 100%	6 h	0 3
Barker et al. [46]	SDD 124	TF	32%	97% BEV	0	100% ProGlide	100%	6 h	1
Zahid et al. [47]	SDD 961 NDD 65814	NR	13% 12%	NR	< 11 625	NR	NR	NR	NR
Litkouhi et al. [48]	SDD 20	TF	NR	33% BEV	0	NR	NR	6 h	0
Memon et al. [25]	SDD 60 NDD 229	TF	4% 13%	48% BEV 41% BEV	0 1	NR	NR	6 h	0 1

Abbreviations: BEV, balloon expandable valve; NDD, next day discharge; SDD, same‐day discharge; TAVI, transcatheter aortic valve implantation; TF, transfemoral.

In most studies, the rate of pre‐existing permanent pacemaker devices did not exceed 13% [25, 42, 43, 45, 47]. There was a predilection for using balloon‐expandable valves [42, 43, 44, 45, 46] however, three studies reported a majority of cases receiving self‐expanding valves [25, 41, 48]. This likely reflects an understanding of the more predictable effects of balloon‐expandable valves on the conduction system [52]. Self‐expanding valves are more likely to require pacemaker implantation, including late pacemaker implantation, than balloon‐expandable valves [53]. Our recent study demonstrated that the electrocardiogram (ECG) at 6 h post‐TAVI did not miss any conduction abnormalities that might have been detected by monitoring up to 24 h [24].

The existing studies on SDD after TAVI don't show any significant increase in mortality or vascular complications [49]. The current breadth of data in TAVI is complemented by similar data in PCI, demonstrating the safety of SDD after cardiac interventions performed via the transfemoral route [23]. The median waiting time for TAVI procedures in the United Kingdom is over 20 weeks, and 299 patients died on the waiting list for TAVI in 2019 alone [8]. Moving toward SDD enhances health services' ability to deliver streamlined care, reduce waiting times, and achieve significant per‐patient cost savings, while raising the potential of increasing capacity for further growth in TAVI procedures [54]. This will also boost patient satisfaction and recovery.

### 1.3 Proposed Pathway for SDD After TAVI

**Figure 2:**
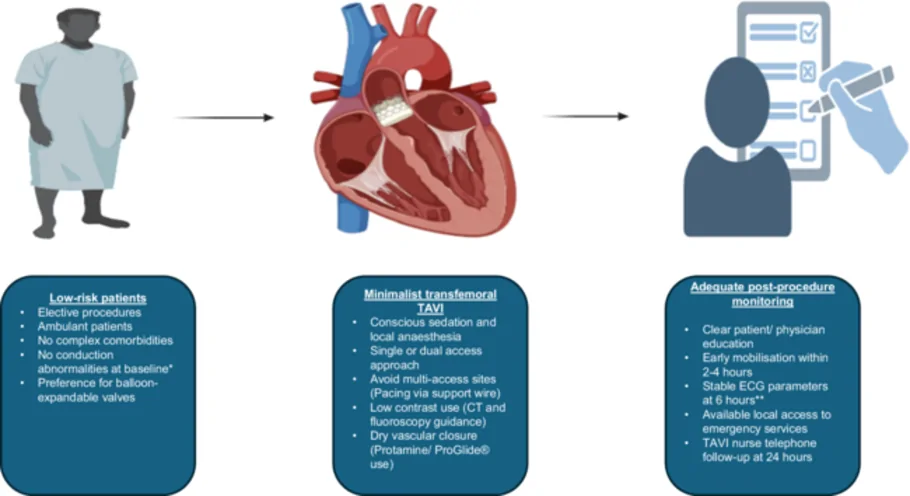
Proposed pathway for same‐day discharge after TAVI. *Defined as sinus node disease, slow atrial fibrillation, right bundle branch block, left bundle branch block with QRS duration of >130 ms, or bifascicular heart block. **Defined as no new heart block, new‐onset persistent left bundle branch block and QRS > 150 ms, PR interval > 240 ms, or a progressive ≥ 20 ms increases in QRS after TAVI. [Color figure can be viewed at wileyonlinelibrary.com]

There are three key components to implementing a successful SDD programme in patients after TAVI. First, the correct selection of patients at low risk of post‐procedure complications (those identified in TAVI outpatient clinics who are undergoing elective procedures without high‐risk clinical features, such as high‐grade heart block on baseline ECG, severe heart failure, coagulopathy, or complex comorbidities). Second, a minimalist TAVI approach that results in optimal angiographic and haemodynamic outcomes. Third, appropriate post‐procedure monitoring and follow‐up with no peri‐procedural complications, a clinically stable condition, normal mobilisation, and a stable 12‐lead ECG at 6 h post‐implant (Figure 2). Other factors associated with patients successfully discharged on the same day after TAVI include clear patient and physician education, a patient's preference for early discharge, and adequate family, geographic, and healthcare support to ensure timely access to emergency care if late complications arise [55]. Careful patient selection, as outlined in Central Illustration 1, is crucial in identifying patients for SDD following TAVI.

**Central illustration 1:**
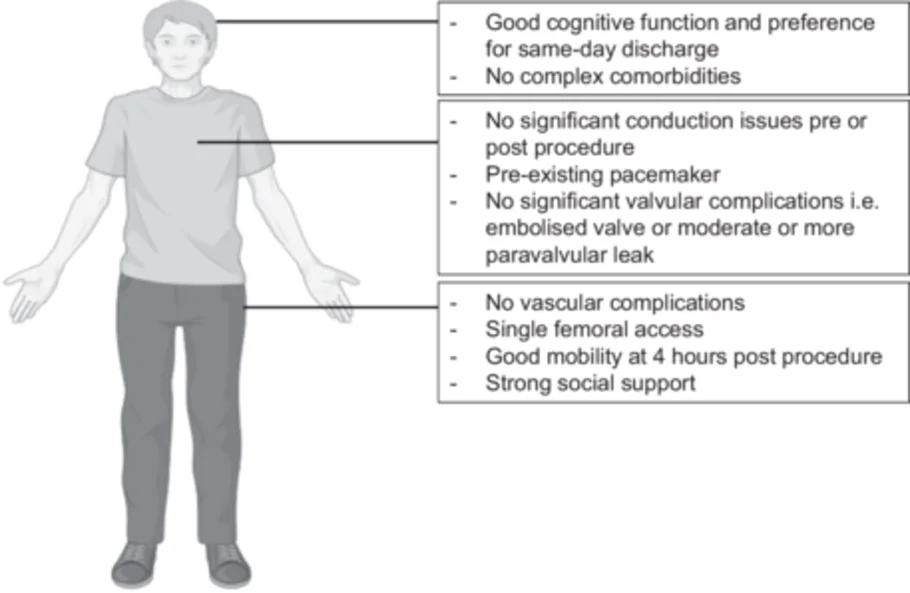
The ideal patient for same‐day discharge following TAVI.

Patients suitable for the standard transfemoral approach and with low‐risk femoral anatomy can be reliably identified on pre‐procedure CT imaging. Furthermore, the ability to exclude adverse aortic root features that may increase the procedure's risk can be detected and assessed. Previous studies of next‐day discharge utilised CT imaging as an essential feature for discharge after TAVI [56]. Similarly, its role in identifying patients for SDD after TAVI remains crucial.

The importance of a minimalist TAVI approach is demonstrated in its ability to reduce complications and facilitate early discharge. Aiming for conscious sedation and local anaesthesia, invasive pressure monitoring via the radial artery or via the primary access‐site sheath in single‐access cases, pacing via the left ventricular support wire and avoiding temporary pacing wires, central lines, and urinary catheters, and using low contrast, all significantly enhance the success of early discharge and reduce complications [56]. It also appears that the use of balloon‐expandable valves, protamine sulfate, and suture‐mediated (e.g., ProGlide) vascular closure devices substantially enhances successful early discharge with minimal vascular complications and/or conduction abnormalities [49]. The BENCHMARK study adopted the minimalist TAVI approach and encouraged early mobilisation to ensure rapid reconditioning post‐procedure [57]. This reduced the length of hospital stay and costs without compromising patient safety.

Cardiac centres implementing the TAVI SDD pathway should ensure that patients are mobilised and monitored within 6 h post‐procedure, and that discharge is not permitted until then. This highlights the crucial role of TAVI specialist nurses in facilitating post‐procedure review and counselling, as well as early telephone follow‐up the day after, which is essential for the widespread roll‐out of SDD after TAVI [56].

## 2 Conclusions

SDD after cardiac interventions has been practised for over 2 decades. There is an increase in waiting times across health services due to the ageing population. There are now multiple observational studies on TAVI demonstrating the safety and practicality of SDD in carefully selected low‐risk patients undergoing elective procedures. There is a need for randomised controlled trials to validate the practice of SDD across TAVI centres.

## Funding

The authors have nothing to report.

## Disclosures

The authors have nothing to report.

## Data Availability

Data sharing not applicable to this article as no datasets were generated or analysed during the current study.
